# Comprehensive lipid and metabolite profiling in healthy adults with low and high consumption of fatty fish: a cross-sectional study – CORRIGENDUM

**DOI:** 10.1017/S0007114522001519

**Published:** 2022-07-28

**Authors:** K. S. Hustad, A. Rundblad, I. Ottestad, J. J. Christensen, K. B. Holven, S. M. Ulven

British Journal of Nutrition/Volume 125/Issue 9/14 May 2021

Published online by Cambridge University Press: 29 June 2020, pp. 1034-1042

Print publication: 14 May 2021

**Details of correction:** reformatted Table 1 supplied

**Existing text:**

See Table 1

**Corrected text should read:**

See updated and reformatted Table 1

**Table 1.** Characteristics of all participants, and participants in the lowest and highest tertiles of fatty fish consumption. (Mean values and standard deviations (SD); median values and quartiles (Q1-Q3); frequencies and percentages)

	All participants		Lowest tertile < 107 g/week		Highest tertile > 223 g/week		
	n=517		n=171		n=171		
	Mean/median	SD/Q1-Q3	Mean/median	SD/Q1-Q3	Mean/median	SD/Q1-Q3	^a^ *P*
**Descriptives**							
Age (years)	55	50-62	55	48-62	57	52-62	0·07
BMI (kg/m^2^)*	25·9	4·0	26·8	4·3	25·6	3·8	<0·001
Sex, n (%)							
Men	225 (44)		64 (37)		92 (54)		<0·05
Women	292 (56)		107 (63)		79 (46)		
Current tobacco user, n (%)†	56 (10·3)		23 (14)		16 (9)		0·30
Lipid lowering therapy, n (%)	26 (5·0)		10 (6)		9 (5)		1
n-3 supplement use, n (%)	259 (50·1)		70 (41)		98 (57)		<0·05
**Blood biochemistry**							
Total cholesterol (mmol/l)‡	5·8	1·1	5·7	1·1	5·9	1·2	0·15
LDL-C (mmol/l)‡	3·7	1·0	3·7	1·0	3·7	1·0	0·44
HDL-C (mmol/l)§	1·6	0·5	1·5	0·4	1·7	0·5	<0·001
Triglycerides (mmol/l)§	1·3	0·7	1·4	0·8	1·3	0·8	0·41
hsCRP, mg/L*	1·1	0·6-2·2	1·3	0·7-2·6	1·0	0·6-1·9	<0·05
**Nutrient intake**							
Fatty fish (g/week)	162	83-255	58	28-82	294	257-372	<0·001
Lean fish (g/week)	108	43-231	68	25-140	172	67-327	<0·001
Energy (kJ/d)	10750	3152	9519	2862	11945	3112	<0·001
Protein (E%)	16·9	2·5	16·4	2·5	17·6	2·7	<0·001
Fat (E%)	35·0	6·0	34·0	6·7	35·7	5·7	<0·05
Saturated (E%)	12·3	2·8	12·5	3·0	12·1	2·6	0·19
Monounsaturated (E%)	12·7	2·6	12·3	3·0	13·0	2·4	<0·05
Polyunsaturated (E%)	6·7	2·2	6·2	2·2	7·2	2·3	<0·001
EPA and DHA (mg/day)	1102	635-1613	498	348-789	1741	1382-2296	<0·001
Carbohydrates (E%)	41·7	6·9	43·5	7·3	39·8	6·9	<0·001
Alcohol (E%)	2·7	1·1-5·5	2·2	0·8-5·2	3·1	1·7-6·0	<0·05

Differences in descriptive variables between low- and high-consumers of fatty fish were tested with t-test for normally distributed variables, Mann-Whitney for variables with skewed distribution and Fisher's exact test for categorical variables. BMI, body mass index; n-3, omega-3; LDL-C, low-density lipoprotein cholesterol; HDL-C, high-density lipoprotein cholesterol; hsCRP, high-sensitivity C-reactive protein; kJ, kilojoule; E%, percentage of total energy intake; EPA, eicosapentaenoic acid; DHA, docosahexaenoic acid.

^a^ *P* for difference between low- and high-consumers of fatty fish. *P*-value <0·05 was considered significant.

* Missing three values, † Missing five values, ‡ Missing one value, § Missing two values

